# Impact of Implementing a Standardized Neonatal Blood Pressure Chart and Neonatal Hypotension Management Guidelines in the Neonatal Intensive Care Unit: A Retrospective Cohort Study

**DOI:** 10.1055/a-2649-1383

**Published:** 2025-07-24

**Authors:** Hanof Bakri, Mohanned Alrahili, Maryam Alkaabi, Mohammed Almahdi, Eman Bazbouz, Rana Almuqati, Manayf Alharbi, Ashwag Alsubaie, Seham Alrsheedi, Amenah A. Essa, Musab Alshareef, Faisal Alsehli, Saif Alsaif, Kamal Ali, Abdulaziz Homedi

**Affiliations:** 1 Department of Neonatal Intensive Care, King Abdulaziz Medical City, Riyadh, Ministry of National Guard Health Affairs, Riyadh, Kingdom of Saudi Arabia; 2 King Abdullah International Medical Research Center, Riyadh, Kingdom of Saudi Arabia; 3 Department of Neonatal Intensive Care, King Saud Bin Abdulaziz University for Health Sciences, Riyadh, Kingdom of Saudi Arabia

**Keywords:** preterm, neonatal hypotension, inotropes, blood pressure

## Abstract

**Objective:**

This study aimed to evaluate the effect of a standardized blood pressure (BP) chart and neonatal hypotension management guidelines on inotrope use and clinical outcomes in preterm infants.

**Study Design:**

Retrospective cohort study of preterm infants (<32 weeks gestational age (GA)) at King Abdulaziz Medical City, Riyadh, Kingdom of Saudi Arabia. We compared data before (EPOCH1) and after (EPOCH2) implementing the BP chart and hypotension guidelines. Extracted variables included maternal/neonatal characteristics, inotrope use, morbidity, and mortality. Statistical significance was set at *p* < 0.05.

**Results:**

Among 384 infants (192 per epoch), overall inotrope use declined significantly in EPOCH2 (33.9 vs. 17.7%, *p* < 0.001). In the hypotension subgroup, EPOCH1 infants had higher GA, birth weight, and 5- and 10-minute APGAR scores. After implementation, dopamine (58.5 vs. 14.7%, *p* < 0.001) and fluid bolus (80 vs. 41.2%, *p* < 0.001) use decreased, whereas norepinephrine (26.2 vs. 70.6%, *p* < 0.001) and hydrocortisone (46.2 vs. 82.4%, *p* < 0.001) increased. Inotrope therapy was triggered at lower mean arterial pressure and systolic/diastolic thresholds; duration of inotrope use also dropped (4.2 vs. 2.6 days, *p* < 0.034). Periventricular leukomalacia rates fell (15.1 vs. 6.5%, *p* < 0.007), with no significant differences in overall mortality, intraventricular hemorrhage, bronchopulmonary dysplasia, retinopathy of prematurity, or necrotizing enterocolitis. However, early mortality (<72 hours) in hypotensive infants was reduced (64.3 vs. 26.7%, *p* < 0.042).

**Conclusion:**

The implementation of BP charts and hypotension management guidelines was associated with a significant reduction in the use of inotropes and fluid boluses, indicating a more standardized and objective approach to BP management in preterm infants. These changes reflect improved clinical decision-making based on defined BP thresholds, resulting in greater consistency in the timing and selection of interventions while potentially minimizing the risks linked to unnecessary cardiovascular support.

**Key Points:**

- Blood pressure charts may improve treatment decisions for neonatal hypotension.
- Hypotension often occurs in preterm infants and can significantly affect their morbidity and mortality.
- Pathophysiology-based management of neonatal hypotension may reduce neonatal morbidity and mortality.

Blood pressure (BP) charts are increasingly used by neonatologists across NICUs to monitor the hemodynamic status of neonates. 1 2 Among the most important factors influencing BP, gestational age (GA) at birth, birth weight (BW), and postmenstrual age (PMA) have been identified as key determinants, reflecting the physiological process of neonatal hemodynamic adaptation to the extrauterine environment and compensatory cardiovascular responses. 3 4 In this context, adequate BP parameters and correct measurement techniques are vital indicators of clinical stability for neonates. 5 6 7 8 Furthermore, several studies define neonatal hypotension using population-based normative values, commonly adopting the fifth or 10th percentile as the threshold for the lowest acceptable BP. 9 10 11 12 13

Neonatal hypotension is a common issue in neonates and carries significant implications for both short- and long-term outcomes. Inotropes are routinely administered to treat low BP or poor perfusion in NICUs. 14 15 Nonetheless, there is a lack of data on the efficacy of antihypotensive treatment, and the thresholds relevant to initiating such treatment remain undefined and require further investigation. 16 Despite extensive research and numerous therapeutic options, neonatologists have yet to reach a consensus on the optimal pharmacological management of neonatal hypotension. 17 18 The challenge for neonatologists working in NICUs is to discern the cause of the hemodynamic changes, determine whether these changes are transitional or pathological, and then proceed to customize the treatment regimen accordingly. 19 20

Neonatal hypotension and rapid fluctuations in BP during the first 72 hours of life in preterm infants are associated with both short- and long-term adverse outcomes, including neurodevelopmental impairment, hearing loss, and increased mortality. 21 Evidence suggests that fluid bolus administration within the first 48 hours may be linked to higher rates of home oxygen dependency, patent ductus arteriosus (PDA), and intraventricular hemorrhage (IVH) among very-low-birth-weight infants. 22 Additionally, treated hypotensive neonates may have lower survival rates or a higher likelihood of surviving with significant morbidity. 23 These hemodynamic disturbances are particularly pronounced in the first week of life in premature infants. 24 However, the absence of high-quality randomized controlled trials addressing key outcomes, coupled with the practical challenges in conducting such studies, continues to hinder our understanding of optimal strategies for the management of neonatal hypotension. 25 26

Therefore, we conducted this study to assess the impact of implementing a standardized BP chart and clinical management guidelines on the treatment of neonatal hypotension. The goal was to support more accurate BP assessment, reduce unnecessary interventions, and potentially minimize associated morbidity and mortality in preterm infants.

## Materials and Methods

This retrospective cohort study involves an examination of preterm infants (<32 weeks of GA) born at King Abdulaziz Medical City, Riyadh, Kingdom of Saudi Arabia. The exclusion criteria included outborn infants and those with birth defects. The project obtained approval from the ethics committee of the King Abdullah International Medical Research Centre (KAIMRC; IRB number: NRR24/059/6).

Data were collected before and after the implementation of the BP charts and neonatal hypotension management guidelines, referred to as EPOCH1 (preimplementation) and EPOCH2 (postimplementation). Information was obtained from an electronic database and included infant demographics, maternal and neonatal characteristics, and the use of inotropic support.

We assessed the following neonatal morbidities: bronchopulmonary dysplasia (BPD) at 36 weeks PMA, major IVH defined as grades III and IV, minor IVH as per Papile classification, necrotizing enterocolitis (NEC) requiring surgical intervention, including intra-abdominal drain insertion, and culture-positive sepsis confirmed in blood, cerebrospinal fluid, or urine. Retinopathy of prematurity (ROP) was also evaluated. In addition, we recorded the duration of respiratory support and the total length of hospital stay until discharge home.

### Implementation of BP Charts and Neonatal Hypotension Management Guidelines

The BP reference values used in this study were based on the Zubrow et al 1 chart, which included 695 infants from 14 NICUs and provides normative values for both transitional (first 24 hours) and posttransitional (beyond 24 hours) periods across a broad GA range. ( Tables 1 and 2 ) This chart remains the most widely referenced in clinical practice for neonatal BP thresholds. To support this approach, we also referenced the study by Elsayed et al, 2 which described similar percentile distributions in 206 infants born at 23 to 28 weeks' gestation. For the first 24 hours of life, a single representative BP measurement was used. This approach was intended to reduce overtreatment during a time when transient hypotension is common due to normal physiological adaptation. Differentiating between physiological and pathological hypotension during this period was critical in guiding management. Our use of this early BP chart was aimed at avoiding unnecessary inotrope use and reducing pressure fluctuations that could contribute to adverse neurological outcomes, particularly in extremely preterm infants.

**Table 1:** Blood pressure values by GA (at birth) for first 24 hours of life

**Table 2:** Blood pressure values by corrected postconceptional age after 24 hours of life

To guide treatment, we used the Zubrow et al BP chart, which defines normative BP percentiles by gestational and postnatal age. The fifth percentile was used as the threshold for initiating therapy for neonatal hypotension, consistent with practices adopted across multiple Canadian NICUs and presented at the 2022 EPIQ collaborative meeting. Clinical signs of neonatal shock prompting evaluation included decreased activity, lethargy, tachycardia or bradycardia, apnea, hypoxemia, prolonged capillary refill time, abnormal temperature regulation (hypothermia or hyperthermia), metabolic acidosis, and elevated serum lactate. Inotropes were weaned and discontinued when three conditions were met: resolution of the primary clinical condition causing hypotension, achievement of stable BP above the 25th percentile, and absence of significant fluctuations or overshoots in mean arterial pressure (MAP; Table 3 ).

**Table 3:** Blood Pressure Values for inotropes weaning after 24 hours of life

The neonatal hypotension management guidelines were developed following an extensive review of the literature, particularly focusing on pathophysiologic causes of hypotension in preterm infants, such as impaired preload, afterload, or contractility, and supported by evidence-based clinical references, including targeted neonatal echocardiography. The development process involved multidisciplinary consensus from neonatologists and senior NICU staff and was approved by the unit's clinical leadership. Educational sessions were conducted for physicians, fellows, nurses, and respiratory therapists prior to rollout to ensure familiarity with the standardized BP chart and clinical algorithm. Implementation commenced in January 2024, and adherence to the guidelines was monitored by prospective documentation of treatment decisions in infants receiving cardiovascular support. Ongoing feedback and clinical audits were used to support the sustained application of the protocol across the unit.

### Statistical Analysis

The data was statistically evaluated using SPSS version 26.0 software. The two-sided tests we applied had significance set at *p* < 0.05. We showcase categorical variables as numbers and percentages, while continuous variables are presented as means and standard deviations (SD). The comparison of categorical variables was done using either Fisher's exact test or the chi-square test. Continuous variables, depending on their distribution, were compared using either the student's *t* -test or the Mann–Whitney U test.

## Results

A total of 384 preterm infants born at less than 32 weeks' gestation were included in the study, with 192 infants in EPOCH1 (before implementation) and 192 in EPOCH2 (after implementation). The mean GA was 29.6 weeks (SD = 2.5) in EPOCH1 and 29.2 weeks (SD = 2.6) in EPOCH2 ( *p* = 0.090). The mean BW was 1,283.1 g (SD = 423) in EPOCH1 and 1,295.5 g (SD = 459) in EPOCH2 ( *p* = 0.784). The mean Apgar score at 5 minutes was 7.9 (SD = 1.1) in EPOCH1 and 7.7 (SD = 1.4) in EPOCH2 ( *p* = 0.177). The proportion of male infants was 47.9% in EPOCH1 and 57.3% in EPOCH2 ( *p* = 0.066), while the proportion of female infants was 52.1% in EPOCH1 and 42.7% in EPOCH2 ( *p* = 0.610). Cesarean section delivery was reported in 159 infants (82.8%) in EPOCH1 and 134 infants (69.8%) in EPOCH2, with this difference reaching statistical significance ( *p* = 0.003). Multiple pregnancies were noted in 76 cases (39.6%) in EPOCH1 and 65 cases (33.9%) in EPOCH2 ( *p* = 0.244). Antenatal steroid administration occurred in 176 infants (91.7%) in EPOCH1 and 180 infants (93.8%) in EPOCH2 ( *p* = 0.432). PROM was present in 60 infants (31.3%) in EPOCH1 and 66 infants (34.4%) in EPOCH2 ( *p* = 0.514). Chorioamnionitis was diagnosed in 18 infants (9.4%) in EPOCH1 and 10 infants (5.2%) in EPOCH2 ( *p* = 0.116). Preeclampsia occurred in 26 mothers (13.5%) in EPOCH1 and 29 mothers (15.1%) in EPOCH2 ( *p* = 0.662). Labetalol use was reported in 19 cases (10%) in EPOCH1 and 26 cases (13.8%) in EPOCH2 ( *p* = 0.258). Early-onset sepsis occurred in eight infants (4.2%) in EPOCH1 and 11 infants (5.7%) in EPOCH2 ( *p* = 0.480), while late-onset sepsis was reported in 22 infants (11.5%) in EPOCH1 and 19 infants (9.9%) in EPOCH2 ( *p* = 0.620). PDA treatment was recorded in 46 infants (24%) in EPOCH1 and 37 infants (19.3%) in EPOCH2, with a statistically significant difference between the two groups ( *p* = 0.004; Table 4 ).

**Table 4:** Overall, the infants' and maternal characteristics

Variables	EPOCH1 (no. 192)	EPOCH2 (no. 192)	*p* -Value
GA (SD)	29.6 (2.5)	29.2 (2.6)	0.090
BW (SD)	1,283.1 (423)	1,295.5 (459)	0.784
Mean APGAR at 5 min (SD)	7.9 (1.1)	7.7 (1.4)	0.177
Gender male	92 (47.9%)	110 (57.3%)	0.066
Gender female	100 (52.1%)	82 (42.7%)	0.610
C-section	159 (82.8%)	134 (69.8%)	0.003
Multiple pregnancy	76 (39.6%)	65 (33.9%)	0.244
Antenatal steroid	176 (91.7%)	180 (93.8%)	0.432
PROM	60 (31.3%)	66 (34.4%)	0.514
Chorioamnionitis	18 (9.4%)	10 (5.2%)	0.116
Preeclampsia	26 (13.5%)	29 (15.1%)	0.662
Labetalol	19 (10%)	26 (13.8%)	0.258
Early sepsis	8 (4.2%)	11 (5.7%)	0.480
Late sepsis	22 (11.5%)	19 (9.9%)	0.620
PDA treatment	46 (24%)	37 (19.3%)	0.004

Abbreviations: BW, birth weight; GA, gestational age; PDA, patent ductus arteriosus; PROM, premature rupture of membrane.

Table 5 presents data on overall neonatal morbidity and mortality. In the EPOCH2 group, the incidence of periventricular leukomalacia (PVL) was significantly lower compared with EPOCH1 (6.5 vs. 15.1%, *p* = 0.007). There were no statistically significant differences observed between the two epochs for BPD (25.1 vs. 24.3%, *p* = 0.856), major IVH (6.3 vs. 3.8%, *p* = 0.112), ROP (4.7 vs. 3.8%, *p* = 0.680), NEC requiring surgery (4.2 vs. 3.7%, *p* = 0.833), duration of invasive ventilation (13.3 vs. 12.2 days, *p* = 0.607), duration of oxygen therapy (31.1 vs. 34.4 days, *p* = 0.333), time to reach full enteral feeds (15.0 vs. 14.8 days, *p* = 0.905), length of hospital stay (51.4 vs. 50.2 days, *p* = 0.749), or mortality rate (8.3 vs. 9.4%, *p* = 0.719; Table 5 ).

**Table 5:** Overall neonatal outcomes of the study population

Variables	EPOCH1 (no. 192)	EPOCH2 (no. 192)	*p* -Value
BPD	48 (25.1%)	45 (24.3%)	0.856
Major IVH	12 (6.3%)	7 (3.8%)	0.112
Minor IVH	62 (32.6%)	47 (25.3%)	
PVL	29 (15.1%)	12 (6.5%)	0.007
ROP	9 (4.7%)	7 (3.8%)	0.680
NEC	8 (4.2%)	7 (3.7%)	0.833
Duration on invasive ventilation in days (SD)	13.3 (17.8)	12.2 (16.9)	0.607
Duration on oxygen in days (SD)	31.1 (32)	34.4 (34.5)	0.333
Time to full feed in days (SD)	15 (13.1)	14.8 (15)	0.905
Length of stay in days (SD)	51.4 (37.5)	50.2 (34.4)	0.749
Mortality	16 (8.3%)	18 (9.4%)	0.719

Abbreviations: BPD, bronchopulmonary dysplasia; IVH, interventricular hemorrhage; NEC, necrotizing enterocolitis; PVL, periventricular leukomalacia; ROP, retinopathy of prematurity.

Table 6 shows a comparison of neonates with hypotension requiring inotropic support between EPOCH1 and EPOCH2. The proportion of infants receiving inotropes was significantly lower in EPOCH2 (33.9 vs. 17.7%, *p* < 0.001). Invasive BP monitoring was more frequently used in EPOCH2 (67.7 vs. 94.1%, *p* = 0.003). GA was higher in EPOCH1 (27.1 vs. 25.3 weeks, *p* = 0.001), as was BW (935.5 g vs. 704.8 g, *p* < 0.001). APGAR scores at 5 and 10 minutes were also higher in EPOCH1 (7.3 vs. 6.2, *p* = 0.001) and (7.6 vs. 6.6, *p* = 0.049), respectively. MAP (27.3 vs. 21 mm Hg, *p* < 0.001), systolic blood pressure (SBP; 39.4 vs. 30.3 mm Hg, *p* < 0.001), and diastolic blood pressure (DBP; 22.2 vs. 15.5 mm Hg, *p* < 0.001) were all significantly higher in EPOCH1. The average duration of inotropic support was longer in EPOCH1 (4.2 vs. 2.6 days, *p* = 0.034). There were no statistically significant differences in the age at initiation of inotropes (3.4 vs. 3.1 days, *p* = 0.844) or in the number of inotropes used (1.87 vs. 2.3, *p* = 0.209); Table 6 ).

**Table 6:** Neonatal hypotension group in EPOCH1 and EPOCH2

Variables	EPOCH1 (no. 65)	EPOCH2 (no. 34)	*p* -Value
Hypotension needs inotropes	65 (33.9%)	34 (17.7%)	<0.001
Invasive BP monitoring	44 (67.7%)	32 (94.1%)	0.003
GA (wk; SD)	27.1 (2.6)	25.3 (1.8)	0.001
BW (g; SD)	935.5 (341)	704.8 (179)	<0.001
APGAR 5 (SD)	7.3 (1.1)	6.2 (1.9)	0.001
APGAR 10 (SD)	7.6 (0.6)	6.6 (1.6)	0.049
MAP (SD)	27.3 (3.9)	21 (2.9)	<0.001
SBP (SD)	39.4 (6.2)	30.3 (5.2)	<0.001
DBP (SD)	22.2 (5.1)	15.5 (2.7)	<0.001
Duration of inotropes in days (SD)	4.2 (4.2)	2.6 (2.4)	0.034
Age starts inotropes in days (SD)	3.4 (6.6)	3.1 (4.7)	0.844
Number of inotropes (SD)	1.87 (1.8)	2.3 (1.3)	0.209

Abbreviations: BW, birth weight; DBP, diastolic blood pressure; GA, gestational age; MAP, mean arterial pressure; SBP, systolic blood pressure.

Table 7 presents the patterns of inotrope and fluid use among hypotensive neonates in EPOCH1 and EPOCH2. Dopamine use significantly declined in EPOCH2 (58.5 vs. 14.7%, *p* < 0.001), while norepinephrine use increased (26.2 vs. 70.6%, *p* < 0.001). Dobutamine administration also rose between epochs (38.5 vs. 64.7%, *p* = 0.013), along with hydrocortisone use (46.2 vs. 82.4%, *p* = 0.001). In terms of fluid support, there was a reduction in the use of normal saline (NS) boluses (80 vs. 41.2%, *p* < 0.001) and a significant increase in the administration of blood products (46.2 vs. 73.5%, *p* = 0.009). No statistically significant differences were observed in the use of epinephrine (20 vs. 35.3%, *p* = 0.096), vasopressin (29.2 vs. 38.2%, *p* = 0.363), or milrinone (3.1 vs. 8.8%, *p* = 0.215; Table 7 ).

**Table 7:** Use of inotropes in EPOCH1 and EPOCH2

Variables	EPOCH1 (no. 65)	EPOCH2 (no. 34)	*p* -Value
Dopamine	38 (58.5%)	5 (14.7%)	<0.001
Norepinephrine	17 (26.2%)	24 (70.6%)	<0.001
Dobutamine	25 (38.5%)	22 (64.7%)	0.013
Epinephrine	13 (20%)	12 (35.3%)	0.096
Vasopressin	19 (29.2%)	13 (38.2%)	0.363
Milrinone	2 (3.1%)	3 (8.8%)	0.215
Hydrocortisone	30 (46.2%)	28 (82.4%)	0.001
Normal saline	52 (80%)	14 (41.2%)	<0.001
Blood products	30 (46.2%)	25 (73.5%)	0.009

Table 8 presents the neonatal outcomes for the subgroup of infants with hypotension. The incidence of BPD was similar between EPOCH1 and EPOCH2 (46.9 vs. 48.1%, *p* = 0.912). No statistically significant differences were noted in the rates of major IVH (19 vs. 13.2%, *p* = 0.563), minor IVH (42.9 vs. 42.9%, *p* = not reported), PVL (23.1 vs. 14.3%, *p* = 0.335), ROP (13.8 vs. 19.2%, *p* = 0.520), or NEC (10.8 vs. 17.2%, *p* = 0.385). Similarly, the duration of invasive ventilation (17.4 vs. 22.6 days, *p* = 0.259), duration on oxygen (53.8 vs. 54.9 days, *p* = 0.907), time to achieve full enteral feeds (23.8 vs. 27.7 days, *p* = 0.461), and length of hospital stay (73.4 vs. 62.4 days, *p* = 0.338) showed no statistically significant differences between the two epochs. However, early mortality within the first 72 hours was significantly lower in EPOCH2 compared with EPOCH1 (64.3 vs. 26.7%, *p* = 0.042; Table 8 ).

**Table 8:** Overall neonatal outcomes of the neonatal hypotension group

Variables	EPOCH1 (no. 65)	EPOCH2 (no. 34)	*p* -Value
BPD	30 (46.9%)	13 (48.1%)	0.912
Major IVH	12 (19%)	3 (13.2%)	0.563
Minor IVH	27 (42.9%)	12 (42.9)	
PVL	15 (23.1%)	4 (14.3%)	0.335
ROP	9 (13.8)	5 (19.2%)	0.520
NEC	7 (10.8%)	5 (17.2%)	0.385
Duration on invasive ventilation in days (SD)	17.4 (20.8)	22.6 (23.1)	0.259
Duration on oxygen in days (SD)	53.8 (40)	54.9 (49.8)	0.907
Time to full feed in days (SD)	23.8 (18)	27.7 (24.5)	0.461
Length of stay in days (SD)	73.4 (51)	62.4 (55.9)	0.338
Mortality < 72 h	9 (64.3%)	4 (26.7%)	0.042

Abbreviations: BPD, bronchopulmonary dysplasia; IVH, interventricular hemorrhage; NEC, necrotizing enterocolitis; PVL, periventricular leukomalacia; ROP, retinopathy of prematurity.

## Discussion

In this retrospective study, the implementation of a BP chart and a neonatal hypotension management guideline was associated with a significant reduction in inotrope usage, suggesting a more standardized and targeted approach to BP management in neonates. Notably, this reduction was observed despite infants in EPOCH2 having lower GA, lower BW, and lower APGAR scores at both 5 and 10 minutes compared with those in the previous period. These findings suggest that the revised management approach may have contributed to improved hemodynamic stability, reducing the perceived need for inotropic support even in a cohort with higher baseline risk factors for hemodynamic instability.

Our data indicate that BP monitoring was more frequently performed using invasive arterial access following the implementation of the new guidelines, improving measurement accuracy and guiding clinical decisions regarding the initiation and discontinuation of inotropes. This approach helped prevent unnecessary interventions and reduced the risk of overtreatment in neonates with neonatal hypotension. In the hypotension group, MAP, SBP, and DBP were lower postimplementation at the time of initiating the therapy. Decisions regarding inotrope initiation and fluid boluses were guided by whether the MAP reached or fell below the fifth percentile, in line with common NICU practices defining neonatal hypotension as MAP < fifth percentile. 27 28 29

Our study demonstrated a significant shift in the management of neonatal hypotension. Initially, dopamine was administered to all neonates with hypotension, regardless of the underlying pathophysiological cause. Following the implementation of the neonatal hypotension management guideline, dopamine use decreased significantly, and treatment decisions became more individualized, focusing on the specific pathophysiological mechanisms of hypotension. 30 31 While dopamine use decreased, norepinephrine use became more common, reflecting a fundamental change in our treatment approach. Unlike dopamine, norepinephrine has minimal effects on pulmonary vascular resistance, making it a more favorable option in certain clinical scenarios. This shift highlights a more targeted approach to managing neonatal hypotension, prioritizing individualized treatment based on underlying pathophysiology. 32 33

Following the implementation of the neonatal hypotension management guideline, we observed a substantial decrease in the use of NS boluses, from 80 to 41%. This reduction aligns with emerging evidence cautioning against the routine use of NS in preterm infants due to its potential associations with adverse outcomes. 34 Studies suggest that excessive NS administration within the first 48 hours of life may contribute to an increased risk of PDA, prolonged dependence on oxygen, and potential long-term neurodevelopmental consequences. 35 36 37 By minimizing unnecessary NS boluses, the revised guideline likely contributed to a more judicious approach to fluid management, reducing the risks associated with excessive volume expansion in this high-risk population. We revised our treatment protocol to include hydrocortisone as a second- or third-line therapy for refractory neonatal hypotension, particularly in cases where initial management strategies, such as fluid resuscitation and inotropes, failed to achieve adequate BP stabilization. This adjustment aligns with evidence supporting the role of corticosteroids in addressing adrenal insufficiency and improving hemodynamic stability in preterm infants with persistent hypotension. 38

In EPOCH 2, we implemented a structured and physiology-based approach to the diagnosis and management of hypotension in preterm infants. Prior to this change, hypotension was defined solely by the traditional threshold of MAP less than or equal to the infant's GA in weeks. This criterion lacked specificity and often resulted in inconsistent clinical responses. To address this, we introduced standardized BP charts and evidence-informed management guidelines that aligned treatment decisions with the underlying pathophysiological mechanisms. The BP chart was divided into three segments: within the first 24 hours of life, after 24 hours, and during the weaning phase once BP exceeded the 25th percentile. The separation between the first 24 hours and the subsequent period allowed differentiation between transitional hypotension, which is frequently physiological, and persistent or pathological hypotension. The management protocol categorized hypotension into two main pathophysiological types. Diastolic hypotension, typically related to low preload or vasodilation, was managed initially with fluid boluses when indicated, followed by vasopressors such as dopamine or norepinephrine. In refractory cases, vasopressin or hydrocortisone was considered. Systolic hypotension, usually associated with poor myocardial contractility or elevated afterload, was managed with inotropes such as epinephrine or dobutamine, with hydrocortisone used selectively based on clinical judgement. A weaning algorithm was also introduced to taper inotropes gradually once the infant's BP had stabilized above the 25th percentile. This approach aimed to minimize the risk of rebound hypotension and reduce the duration of inotropic support. This transition to a physiology-driven and standardized framework allowed for more targeted intervention, and we believe it contributed to the observed reduction in early mortality without increasing late mortality or the overall use of inotropes. This structured approach may be replicable or adaptable by other neonatal units seeking to improve the consistency and effectiveness of BP management in preterm infants.

In our study, the rates of major neonatal morbidities, including BPD, major and minor IVH, PVL, ROP, and NEC, did not significantly differ between EPOCH1 and EPOCH2 in the hypotension group. Additionally, there was no significant difference in the duration of invasive ventilation, total oxygen dependence, time to achieve full enteral feeding, or overall length of hospital stay between the two epochs. These findings suggest that while the implementation of the neonatal hypotension management guideline standardized treatment approaches, it did not lead to notable changes in the incidence of these complications. The similarity in morbidity rates may be attributed to the fact that factors influencing these outcomes, such as prematurity-related lung disease, hemodynamic stability, and neonatal intensive care practices, remained consistent between the two periods. Further research is needed to explore whether refinements in neonatal BP management strategies could contribute to improved long-term outcomes. Although overall mortality remained unchanged between the two epochs, early mortality within 72 hours was significantly lower in EPOCH2. We did not observe a corresponding increase in late mortality after inotrope exposure. Review of individual cases did not suggest a pattern linking inotrope therapy to subsequent death, and late mortality appeared attributable to multifactorial complications of extreme prematurity. Nevertheless, larger prospective studies are needed to better assess the relationship between inotrope use and late mortality.

## Strengths and Limitations

Our study has several strengths and limitations. One of the primary limitations is its retrospective design, as data for both maternal and neonatal variables were collected from medical records, which may be subject to missing or incomplete information. Additionally, this study was conducted at a single institution, which may restrict the generalizability of the findings to other neonatal units with different patient populations, clinical protocols, and resource availability. Despite these limitations, the study has notable strengths. It provides a systematic comparison of neonatal mortality and major morbidities before and after implementing standardized BP monitoring and management guidelines. The use of a clearly defined cohort of preterm infants allows for a focused evaluation of the impact of these interventions on neonatal outcomes.

## Conclusion

The implementation of BP charts and hypotension management guidelines was associated with a significant reduction in the use of inotropes and fluid boluses, indicating a more standardized and objective approach to BP management in preterm infants. These changes reflect improved clinical decision-making based on defined BP thresholds, resulting in greater consistency in the timing and selection of interventions while potentially minimizing the risks linked to unnecessary cardiovascular support.

Despite these improvements, further investigations are warranted to assess the long-term clinical implications of this approach. Specifically, future studies should evaluate whether reduced inotrope and fluid administration translates into improved neurodevelopmental outcomes, reduced incidence of complications such as IVH or BPD, and overall survival benefits. Additionally, ongoing efforts should focus on refining BP chart parameters to ensure their applicability across diverse neonatal populations while minimizing the risks of both over- and under-treatment. Establishing the safest and most effective BP monitoring and management strategies will be crucial in optimizing outcomes for preterm neonates in the NICU setting.

## Notes

**What Is Already Known on This Topic**

- Hypotension in preterm infants is a common problem, with significant implications for morbidity and mortality.
- Inotropes are often used to manage neonatal hypotension in neonatal intensive care units. The literature defines neonatal hypotension as the lowest acceptable BP according to the fifth or 10th percentile of population-based normative values.
- In neonatal hypotension, significant short- and long-term morbidity is associated with the rapid fluctuations in BP observed after the initiation of inotropes in preterm infants.

**What This Study Adds**

- Standardized BP charts have improved therapeutic decision-making for the treatment of neonatal hypotension by providing accurate information and preventing unnecessary treatment.
- Approaches based on pathophysiology for managing neonatal hypotension may assist in reducing the number and duration of inotrope use.

## References

[1] Philadelphia Neonatal Blood Pressure Study Group Zubrow A B Hulman S Kushner H Falkner B Determinants of blood pressure in infants admitted to neonatal intensive care units: a prospective multicenter study J Perinatol 1995 15 06 470 479 8648456

[2] Elsayed Y Ahmed F Blood pressure normative values in preterm infants during postnatal transition Pediatr Res 2024 95 03 698 704 37667035 10.1038/s41390-023-02788-8

[3] Dore R Barnes K Bremner S Neonatal blood pressure by birth weight, gestational age, and postnatal age: a systematic review Matern Health Neonatol Perinatol 2024 10 01 9 38689326 10.1186/s40748-024-00180-w PMC11061963

[4] Dionne J M Determinants of blood pressure in neonates and infants: predictable variability Hypertension 2021 77 03 781 787 33566691 10.1161/HYPERTENSIONAHA.120.14587

[5] International Neonatal Consortium Dionne J M Bremner S A Baygani S K Method of blood pressure measurement in neonates and infants: a systematic review and analysis J Pediatr 2020 221 23 3.1E6 32446487 10.1016/j.jpeds.2020.02.072

[6] Oliveira Blackman A Oliveira G SO Melo J OM Estimation of blood pressure levels in healthy newborns Eur Heart J 2023 44 02 233237316965

[7] Batton B Neonatal Blood Pressure Standards: What Is “Normal”? Clin Perinatol 2020 47 03 469 485 32713445 10.1016/j.clp.2020.05.008

[8] Nuntnarumit P Yang W Bada-Ellzey H S Blood pressure measurements in the newborn Clin Perinatol 1999 26 04 981 996 10572732

[9] Noori S Seri I Evidence-based versus pathophysiology-based approach to diagnosis and treatment of neonatal cardiovascular compromise Semin Fetal Neonatal Med 2015 20 04 238 245 25823937 10.1016/j.siny.2015.03.005

[10] Pereira S S Sinha A K Morris J K Wertheim D F Shah D K Kempley S T Blood pressure intervention levels in preterm infants: pilot randomised trial Arch Dis Child Fetal Neonatal Ed 2019 104 03 F298 F305 30049724 10.1136/archdischild-2017-314159

[11] Lalan S P Warady B A Discrepancies in the normative neonatal blood pressure reference ranges Blood Press Monit 2015 20 04 171 177 25756223 10.1097/MBP.0000000000000116

[12] Samanta M Mondal R Ray S Normative blood pressure data for Indian neonates Indian Pediatr 2015 52 08 669 673 26388624 10.1007/s13312-015-0694-y

[13] Dilli D Soylu H Tekin N Neonatal hemodynamics and management of hypotension in newborns Turk Pediatri Ars 2018 53 01 S65 S75 31236020 10.5152/TurkPediatriArs.2018.01801 PMC6568285

[14] Ruoss J L McPherson C DiNardo J Inotrope and vasopressor support in neonates Neoreviews 2015 16 06 e351 e61

[15] Noori S Seri I Neonatal blood pressure support: the use of inotropes, lusitropes, and other vasopressor agents Clin Perinatol 2012 39 01 221 238 22341548 10.1016/j.clp.2011.12.010

[16] Persad E Brindefalk B Rakow A Blood pressure trends following birth in infants born under 25 weeks' gestational age: a retrospective cohort study BMJ Paediatr Open 2024 8 01 e002438 10.1136/bmjpo-2023-002438 PMC10966797 38531550

[17] Verma R P Dasnadi S Zhao Y Chen H H Complications associated with the current sequential pharmacological management of early postnatal hypotension in extremely premature infants Proc Bayl Univ Med Cent 2019 32 03 355 360 31384186 10.1080/08998280.2019.1585732 PMC6650250

[18] Osborn D A Paradisis M Evans N The effect of inotropes on morbidity and mortality in preterm infants with low systemic or organ blood flow Cochrane Database Syst Rev 2007 2007 01 CD005090 17253539 10.1002/14651858.CD005090.pub2 PMC8860620

[19] Joynt C Cheung P Y Treating hypotension in preterm neonates with vasoactive medications Front Pediatr 2018 6 86 29707527 10.3389/fped.2018.00086 PMC5908904

[20] Pereira S S Sinha A K Shah D K Kempley S T Variation between clinician-recorded and downloaded invasive blood pressure in extremely preterm infants Am J Perinatol 2024 41(S 01):e1756 e1758 37040879 10.1055/a-2071-3145

[21] Fanaroff A A Fanaroff J M Short- and long-term consequences of hypotension in ELBW infants Semin Perinatol 2006 30 03 151 155 16813974 10.1053/j.semperi.2006.04.006

[22] Bakshi S Koerner T Knee A Singh R Vaidya R Effect of fluid bolus on clinical outcomes in very low birth weight infants J Pediatr Pharmacol Ther 2020 25 05 437 444 32641914 10.5863/1551-6776-25.5.437 PMC7337138

[23] Batton B Batton D Riggs T Blood pressure during the first 7 days in premature infants born at postmenstrual age 23 to 25 weeks Am J Perinatol 2007 24 02 107 115 17304424 10.1055/s-2007-970178

[24] Pejovic B Peco-Antic A Marinkovic-Eric J Blood pressure in non-critically ill preterm and full-term neonates Pediatr Nephrol 2007 22 02 249 257 17053885 10.1007/s00467-006-0311-3

[25] Garvey A A Kooi E MW Dempsey E M Inotropes for preterm infants: 50 years on are we any wiser? Front Pediatr 2018 6 88 29682496 10.3389/fped.2018.00088 PMC5898425

[26] HIP consortium Dempsey E M Barrington K J Marlow N Hypotension in preterm infants (HIP) randomised trial Arch Dis Child Fetal Neonatal Ed 2021 106 04 398 403 33627329 10.1136/archdischild-2020-320241 PMC8237176

[27] Eunice Kennedy Shriver National Institute of Child Health & Human Development Neonatal Research Network Batton B Li L Newman N S Use of antihypotensive therapies in extremely preterm infants Pediatrics 2013 131 06 e1865 e1873 23650301 10.1542/peds.2012-2779 PMC3666108

[28] Alderliesten T Lemmers P M van Haastert I C Hypotension in preterm neonates: low blood pressure alone does not affect neurodevelopmental outcome J Pediatr 2014 164 05 986 991 24484771 10.1016/j.jpeds.2013.12.042

[29] Giesinger R E Levy P T Ruoss J L Cardiovascular management following hypoxic-ischemic encephalopathy in North America: need for physiologic consideration Pediatr Res 2021 90 03 600 607 33070162 10.1038/s41390-020-01205-8 PMC8249436

[30] Eunice Kennedy Shriver National Institute of Child Health and Human Development Neonatal Research Network Batton B Li L Newman N S Evolving blood pressure dynamics for extremely preterm infants J Perinatol 2014 34 04 301 305 24503912 10.1038/jp.2014.6 PMC3982788

[31] Giesinger R E McNamara P J Hemodynamic instability in the critically ill neonate: an approach to cardiovascular support based on disease pathophysiology Semin Perinatol 2016 40 03 174 188 26778235 10.1053/j.semperi.2015.12.005

[32] Ishiguro A Suzuki K Sekine T Effect of dopamine on peripheral perfusion in very-low-birth-weight infants during the transitional period Pediatr Res 2012 72 01 86 89 22441378 10.1038/pr.2012.37

[33] McNamara P J Giesinger R E Lakshminrusimha S Dopamine and neonatal pulmonary hypertension-pressing need for a better pressor? J Pediatr 2022 246 242 250 35314154 10.1016/j.jpeds.2022.03.022

[34] Sehgal A Gauli B Changes in respiratory mechanics in response to crystalloid infusions in extremely premature infants Am J Physiol Lung Cell Mol Physiol 2023 325 06 L819 L825 37933458 10.1152/ajplung.00179.2023

[35] Aslam A Vincer M Allen A Imanullah S O'Connell C M Long-term outcomes of saline boluses in very preterm infants J Neonatal Perinatal Med 2018 11 03 317 321 30040744 10.3233/NPM-17105

[36] Lawn C J Weir F J McGuire W Base administration or fluid bolus for preventing morbidity and mortality in preterm infants with metabolic acidosis Cochrane Database Syst Rev 2005 2005 02 CD003215 15846651 10.1002/14651858.CD003215.pub2 PMC8711593

[37] Tyler W Ewer A K The use of volume expansion in preterm infants Paediatr Perinat Epidemiol 2004 18 02 135 137 14996253 10.1111/j.1365-3016.2003.00543.x

[38] Mizobuchi M Yoshimoto S Nakao H Time-course effect of a single dose of hydrocortisone for refractory hypotension in preterm infants Pediatr Int 2011 53 06 881 886 21429058 10.1111/j.1442-200X.2011.03372.x

